# Predicting and Understanding Cancer Response to Treatment

**DOI:** 10.1155/2018/6159214

**Published:** 2018-05-29

**Authors:** Maurizio Callari, Paolo Gandellini, Ira Skvortsova, Paul N. Span

**Affiliations:** ^1^Cancer Research UK Cambridge Institute, University of Cambridge, Cambridge, UK; ^2^Fondazione IRCCS Istituto Nazionale dei Tumori, Milan, Italy; ^3^Innsbruck Medical University, Innsbruck, Austria; ^4^Radiotherapy & OncoImmunology Laboratory, Department of Radiation Oncology, Radboud University Medical Center, Nijmegen, Netherlands

Cancer treatment is increasingly based on the use of drugs targeting specific genes or pathways. This is the result of years of cancer research aiming at identifying suitable targets as well as highly selective and effective compounds. However, strong single gene-single drug pharmacogenomics associations are rarely observed, and it is becoming clear that cancer response or resistance to treatment underlies much more complex mechanisms.

Understanding such mechanisms is key to developing predictive biomarkers able to identify which patients are likely to respond to a specific treatment. Unfortunately, in most clinical scenarios, there is a lack of adequate predictive biomarkers. As a consequence, a large proportion of patients are either overtreated or receive ineffective treatments, challenging the effective implementation of a precision medicine.

We set up this special issue with such a framework in mind, seeking for original research papers and reviews giving insights into the latest advances in the field. The series of included manuscripts clearly highlights how vast and heterogeneous the definition of predictive biomarkers can be. It could be measured in different tissue types (e.g., tumour or liquid biopsies) and could be any entity besides the standard clinicopathological parameters, ranging from genetic mutations to epigenetic and metabolic changes.

P. Bossi et al. in “Are Fusion Transcripts in Relapsed/Metastatic Head and Neck Cancer Patients Predictive of Response to Anti-EGFR Therapies?” investigated the predictive role of fusion transcripts in head and neck squamous cell carcinoma treated with chemotherapy and cetuximab, identifying the CD274-PDCD1LG2 fusion as enriched in short-PFS patients and associated with worse survival within this subgroup.

N. Bedini et al. in “Evaluation of Mediators Associated to the Inflammatory Response in Prostate Cancer Patients Undergoing Radiotherapy” explored the hypothesis that a previous surgery may influence plasma level of inflammatory molecules in prostate cancer patients, resulting in enhanced radiosensitivity. The levels of six inflammation mediators were measured in a cohort of prostate cancer patients undergoing radical radiotherapy, and CCL2 levels were found to be significantly higher in patients experiencing grade 2 toxicity.

There is a growing interest in developing noninvasive approaches to monitor cancer progression and response to treatment. M. Verduin et al. thoroughly describe the state-of-art for glioblastoma in the review titled “Noninvasive Glioblastoma Testing: Multimodal Approach to Monitoring and Predicting Treatment Response.” In this review, the authors discuss multiple approaches and their effect on predicting and monitoring treatment response in glioblastoma. This set of diagnostic approaches comprises advanced MRI techniques, nuclear imaging, liquid biopsy, and new integrated approaches including radiogenomics and radiomics.

Moving to breast cancer, a crucial aspect of this disease is its extensive heterogeneity, definitely demonstrated at genomics, transcriptomics, and proteomics level. V. Cappelletti et al. in “Metabolic Footprints and Molecular Subtypes in Breast Cancer” clearly show that such heterogeneity is present also at the metabolic level. After a brief overview of the literature on molecular subtypes and an account of major metabolic pathways in cancer, original metabolomics data from a series of primary breast cancer patients are reported. Intriguingly, the luminal B subgroup represents a tumour type that preferentially relies on fatty acids for energy, whereas HER2 and basal-like ones prevalently show alterations in glucose/glutamine metabolism. This could enable the development of new breast cancer subtype-specific therapeutic strategies and associated biomarkers.

M. Aubele et al. in “The Predictive Value of *PITX2* DNA Methylation for High-Risk Breast Cancer Therapy: Current Guidelines, Medical Needs, and Challenges” focused on the triple-negative subtype of breast cancer that, together with cases having more than three positive lymph nodes, constitutes a high-risk group for which guidelines recommend anthracycline-based chemotherapy as the standard of care. However, only a proportion of patients benefit from this treatment and methylation of the PITX2 (paired-like homeodomain transcription factor 2) gene might serve as a novel predictive biomarker. This review specifically discusses the future clinical application of PITX2 as a predictive biomarker to personalize breast cancer management.

Finally, M. Di Modica et al. in “Predicting the Efficacy of HER2-Targeted Therapies: A Look at the Host” draw their attention on another breast cancer subtype, HER2-positive tumours, for which a targeted therapy is available, that is, trastuzumab/Herceptin, a monoclonal antibody targeting HER2. However, a proportion of patients do not respond to this agent, whereas new drugs have proven to be efficacious in clinical trials. Biomarker-based stratification of the HER2-positive subtype is therefore needed, and in this review, the authors discuss in particular the role of the immune system in shaping the response.

Overall, manuscripts in this issue highlighted how extremely different aspects of the tumour and of the host can impact and determine response or resistance to treatment. Future research efforts should aim to a multidimentional characterization of both the tumour and the host/microenvironment and to the development of data integration strategies. This is probably the best way to capture the complexity behind cancer response to treatment and develop predictive biomarkers able to reach the high accuracy required for their clinical implementation.

*Maurizio Callari* *Paolo Gandellini* *Ira Skvortsova* *Paul N. Span*

